# Comparative diagnostic value of a blood-based three-gene DNA methylation assay and a seven-autoantibody panel for differentiating pulmonary nodules

**DOI:** 10.1016/j.jlb.2026.100498

**Published:** 2026-09-10

**Authors:** Dong Rui, Xunsi Liang, Min Yi, Shaowei Zhan, Shui Xiang, Jifei Chen, Jiachen Hou, Xin Wu, Xuejun Qin, Zhougui Ling

**Affiliations:** a Department of Respiratory and Critical Care Medicine, The Fourth Affiliated Hospital of Guangxi Medical University, Liuzhou, Guangxi, China; b Department of Thoracic Surgery, Chest Hospital of Guangxi Zhuang Autonomous Region, Liuzhou, Guangxi, China; c The Pathology Department of the Fourth Affiliated Hospital of Guangxi Medical University, Liuzhou, Guangxi, China; d Department of Thoracic Surgery, The Fourth Affiliated Hospital of Guangxi Medical University, Liuzhou, Guangxi, China; e Department of Medical Imaging, The Fourth Affiliated Hospital of Guangxi Medical University, Liuzhou, Guangxi, China

**Keywords:** Pulmonary nodule, Lung cancer, DNA methylation, Tumour-associated autoantibody, Liquid biopsy

## Abstract

**Background:**

Low-dose CT has increased pulmonary nodule detection, but noninvasively distinguishing benign from malignant nodules or early lung cancer remains difficult. Blood-based DNA methylation and tumour-associated autoantibody assays may complement imaging.

**Methods:**

This combined prospective and retrospective study evaluated two blood-based strategies. The prospective methylation cohort (141 participants; May 2024–April 2026) comprised 91 malignant, 22 benign-nodule, and 28 high-risk individuals, with plasma *SHOX2*, *RASSF1A*, and *PTGER4* methylation measured by multiplex fluorescence PCR. The retrospective cohort (446 participants; January 2023–April 2025) comprised 193 malignant, 118 benign-nodule, and 135 high-risk individuals, with serum conventional markers (CYFRA21-1, CEA, NSE, SCC) and seven autoantibodies (p53, GAGE7, PGP9.5, CAGE, MAGEA1, SOX2, GBU4-5) analysed. Performance was assessed by positive rate, sensitivity, specificity, AUC, and Youden index.

**Results:**

Positive rates in the malignant versus benign versus high-risk groups were 83.52% vs 45.45% vs 10.71% for the three-methylation panel and 63.20% vs 16.95% vs 10.37% for the seven-autoantibody panel (both *P* < 0.05). In early-stage (I–II) disease, the methylation panel had the highest positive rate (83.87%), exceeding the autoantibody panel (67.53%) and conventional markers (37.66%; *P* < 0.05), whereas conventional markers were highest in advanced (III–IV) disease (93.97%). Early-stage AUCs were 0.789 for methylation (sensitivity 83.87%, specificity 74.00%), 0.771 for autoantibodies (sensitivity 67.53%, specificity 86.56%), and 0.570 for conventional markers, rising to 0.851 in advanced disease. Combining the methylation and autoantibody panels raised early-stage sensitivity to 94.75% (AUC 0.794). As additional clinically oriented validation, the methylation panel yielded an overall positive predictive value of 85.39%, negative predictive value of 71.15%, accuracy of 80.14%, positive likelihood ratio of 3.21, and negative likelihood ratio of 0.22 for malignant disease versus all controls.

**Conclusions:**

Both panels aided differentiation of malignant from benign pulmonary nodules; methylation gave the strongest early-stage sensitivity and autoantibodies higher specificity, both outperforming conventional markers. Combined testing may improve sensitivity but requires multicentre validation.

## 1 Introduction

Lung cancer remains the leading cause of cancer mortality worldwide. GLOBOCAN 2022 estimated approximately 2.48 million new lung cancer cases and 1.82 million deaths globally, and recent national data from China also identify lung cancer as a dominant contributor to cancer incidence and mortality [1,2]. Prognosis is strongly stage dependent: patients with adenocarcinoma in situ or minimally invasive adenocarcinoma can achieve excellent long-term survival after complete resection, whereas advanced-stage disease continues to have substantially poorer outcomes [3].

Low-dose computed tomography (LDCT) screening reduces lung cancer mortality in high-risk populations, as demonstrated by the National Lung Screening Trial [4]. However, LDCT also detects many indeterminate pulmonary nodules, most of which are benign. Distinguishing malignant nodules from inflammatory, infectious, and other benign lesions remains a common clinical problem. Overdiagnosis, short-interval surveillance, invasive biopsy, and delayed treatment are all potential consequences of uncertainty in nodule evaluation.

In current clinical practice, CT-based assessment remains the diagnostic foundation for pulmonary nodules. Structured imaging interpretation and risk models use clinical and radiologic variables such as age, smoking history, nodule size, upper-lobe location, attenuation, multiplicity, and spiculation to estimate malignancy probability [5,6]. Therefore, a blood-based methylation assay should be interpreted as an adjunct to, rather than a replacement for, LDCT and multidisciplinary nodule evaluation.

Liquid biopsy offers a minimally invasive route to molecular risk assessment. DNA methylation is an early and recurrent epigenetic event in carcinogenesis [7,8]. Several studies have reported diagnostic value for *SHOX2*, *RASSF1A*, and *PTGER4* methylation in plasma, bronchial aspirates, bronchoalveolar lavage fluid, and other biospecimens [[9], [10], [11], [12], [13], [14]]. These markers are attractive because methylation changes may become detectable before an advanced tumour burden develops.

Tumour-associated autoantibodies represent another complementary blood-based strategy. Autoantibodies against tumour antigens can arise during early tumour–host immune interactions and may be relatively stable in serum. Panels including p53, PGP9.5, SOX2, GAGE7, GBU4-5, MAGEA1, and CAGE have been studied for early lung cancer detection and for discriminating malignant from benign pulmonary nodules [[15], [16], [17], [18], [19]]. Nevertheless, direct comparisons between DNA methylation and autoantibody panels in pulmonary nodule populations remain limited.

This study compared a plasma *SHOX2*/*RASSF1A*/*PTGER4* methylation panel with a serum seven-autoantibody panel and conventional serum tumour markers for differentiating benign and malignant pulmonary nodules, with emphasis on early-stage lung cancer.

## 2 Materials and methods

### 2.1 Study design and participants

This was a combined prospective and retrospective diagnostic study. For the prospective methylation cohort, participants were enrolled between May 2024 and April 2026 from Liuzhou Workers' Hospital/The Fourth Affiliated Hospital of Guangxi Medical University and Guangxi Chest Hospital. The cohort included 91 patients with malignant pulmonary nodules or lung cancer, 22 patients with benign pulmonary nodules, and 28 high-risk individuals undergoing health examination. For the retrospective autoantibody cohort, records from Liuzhou Workers' Hospital between January 2023 and April 2025 were reviewed, including 193 malignant cases, 118 benign pulmonary nodule controls, and 135 high-risk controls.

Eligible participants were at least 18 years old and had not received surgery, radiotherapy, chemotherapy, immunotherapy, or targeted therapy before blood sampling. Benign pulmonary nodules were defined as focal pulmonary lesions 3 cm or smaller that were shown to be nonmalignant by pathology or that resolved or regressed after anti-infective treatment and follow-up. Early-stage malignant pulmonary nodules or lung cancers were defined as pathologically confirmed stage I–II disease; stage III–IV disease was classified as locally advanced or advanced. Staging followed contemporary IASLC/UICC TNM principles [20], and histological categories were interpreted according to contemporary lung tumour classification principles where applicable [21].

High-risk controls were selected according to smoking and risk-factor criteria, including older heavy smokers and individuals with additional risk factors such as prior cancer history, chronic lung disease, family history of lung cancer, radon exposure, occupational exposure, emphysema, or chronic obstructive pulmonary disease. Exclusion criteria included prior malignancy with recurrence or metastasis risk within five years, severe trauma or major acute cardiovascular or cerebrovascular events within one month, pregnancy or lactation, severe hepatic or renal dysfunction, long-term haemodialysis or severe coagulation disorders affecting sample quality, and participation in another interventional clinical trial.

The study was approved by the Ethics Committee of Liuzhou Workers' Hospital (approval number KY2025472,KY2024005). Consent procedures followed the approved protocol.

### 2.2 Biomarker assays

For the seven-autoantibody panel, fasting venous blood was collected in serum tubes. After standing at room temperature for 1–2 h, samples were centrifuged at 3000 rpm for 15 min, and serum was stored at −80 °C until testing. The panel measured serum autoantibodies against p53, PGP9.5, SOX2, GAGE7, GBU4-5, MAGEA1, and CAGE using an enzyme-linked immunosorbent assay (ELISA) according to the manufacturer's instructions. The test was considered positive when one or more autoantibodies exceeded the reference limit: p53 13.09 U/mL, PGP9.5 11.10 U/mL, SOX2 10.26 U/mL, GAGE7 14.36 U/mL, GBU4-5 6.99 U/mL, MAGEA1 11.92 U/mL, or CAGE 7.23 U/mL.

For DNA methylation testing, 10 mL of fasting venous blood was collected in EDTA-K2 anticoagulant tubes. Plasma was separated immediately or stored at 2–8 °C for no longer than 4 h before separation, and separated plasma was stored at −80 °C. Cell-free DNA was extracted, bisulfite converted, and tested by multiplex fluorescence PCR targeting methylated *SHOX2*, *RASSF1A*, and *PTGER4* sequences. *ACTB* served as the internal reference. A valid sample required a mean *ACTB* Ct value of 35.0 or lower. A sample was classified as methylation positive when the sample *P* value was 2.0 or higher and negative when the sample *P* value was below 2.0.

Conventional tumour markers, including CYFRA21-1, CEA, NSE, and SCC antigen, were measured by electrochemiluminescence immunoassay. The conventional tumour marker panel was classified as positive if any marker exceeded its clinical reference limit: CYFRA21-1 3.3 ng/mL, CEA 5.0 ng/mL, NSE 16.3 ng/mL, or SCC antigen 2.7 ng/mL.

### 2.3 Blinding and bias control

Sample collection and laboratory testing were performed by separate trained personnel. Laboratory staff and statistical analysts were blinded to clinical diagnosis and staging information during testing and data processing.

### 2.4 Statistical analysis

Continuous variables are presented as mean ± standard deviation and categorical variables as number and percentage. Group comparisons used analysis of variance or t tests for continuous variables and chi-square (*χ*^*2*^) tests for categorical variables, as appropriate. Diagnostic performance was evaluated using receiver operating characteristic (ROC) curves, the area under the ROC curve (AUC), sensitivity, specificity, and the Youden index. Sensitivity and specificity were calculated according to the assay cutoff value. Two-sided *P* values below 0.05 were considered statistically significant. Analyses were performed with SPSS version 27.0. To strengthen clinical interpretation, we additionally calculated positive predictive value, negative predictive value, accuracy, and likelihood ratios for the three-DNA-methylation panel using malignant cases as the disease group and benign nodules plus high-risk individuals as controls.

## 3 Results

### 3.1 Baseline characteristics

In the methylation cohort, age, sex, and smoking status were comparable among the malignant, benign nodule, and high-risk groups (all *P* > 0.05). The malignant group included 45 men and 46 women, with a mean age of 63.57 ± 9.98 years. The benign nodule group included 10 men and 12 women, with a mean age of 60.05 ± 11.72 years. The high-risk group included 14 men and 14 women, with a mean age of 61.21 ± 10.71 years.

The retrospective autoantibody cohort also showed balanced baseline features. The malignant group had a mean age of 58.81 ± 9.91 years, the benign nodule group 57.92 ± 10.62 years, and the high-risk group 57.31 ± 11.22 years. Sex distribution and smoking status did not differ significantly among the groups (Table 1).

**Table 1:** Baseline characteristics of the two analytic cohorts.

Cohort and variable	Malignant	Benign nodule	High-risk	P value
**DNA methylation cohort, n**	**91**	**22**	**28**	
Age, years	63.57 ± 9.98	60.05 ± 11.72	61.21 ± 10.71	0.275
Male sex, n (%)	45 (49.45)	10 (45.45)	14 (50.00)	0.937
Smoker, n (%)	50 (54.95)	13 (59.10)	15 (53.57)	0.920

**Seven-autoantibody cohort, n**	**193**	**118**	**135**	

Age, years	58.81 ± 9.91	57.92 ± 10.62	57.31 ± 11.22	0.433
Male sex, n (%)	141 (73.06)	84 (71.19)	97 (71.85)	0.932
Smoker, n (%)	128 (66.32)	72 (61.02)	73 (54.07)	0.081

Values are mean ± standard deviation or n (%). *P* values compare the three groups within each cohort.

### 3.2 Positive rates by group, stage, and histology

The three-DNA-methylation panel was positive in 76 of 91 malignant cases (83.52%), higher than in benign nodules (10 of 22; 45.45%) and high-risk controls (3 of 28; 10.71%) (*P* < 0.05; Fig. 1A). Within malignant cases, the positive rate was similar between stage I–II disease (52 of 62; 83.87%) and stage III–IV disease (24 of 29; 82.76%) (*P* > 0.05; Fig. 1B). By histology, methylation was positive in 59 of 74 adenocarcinomas (79.73%), all 6 squamous cell carcinomas, both small cell lung cancers, and all 9 other malignant tumours (Fig. 1C).

**Fig. 1:**
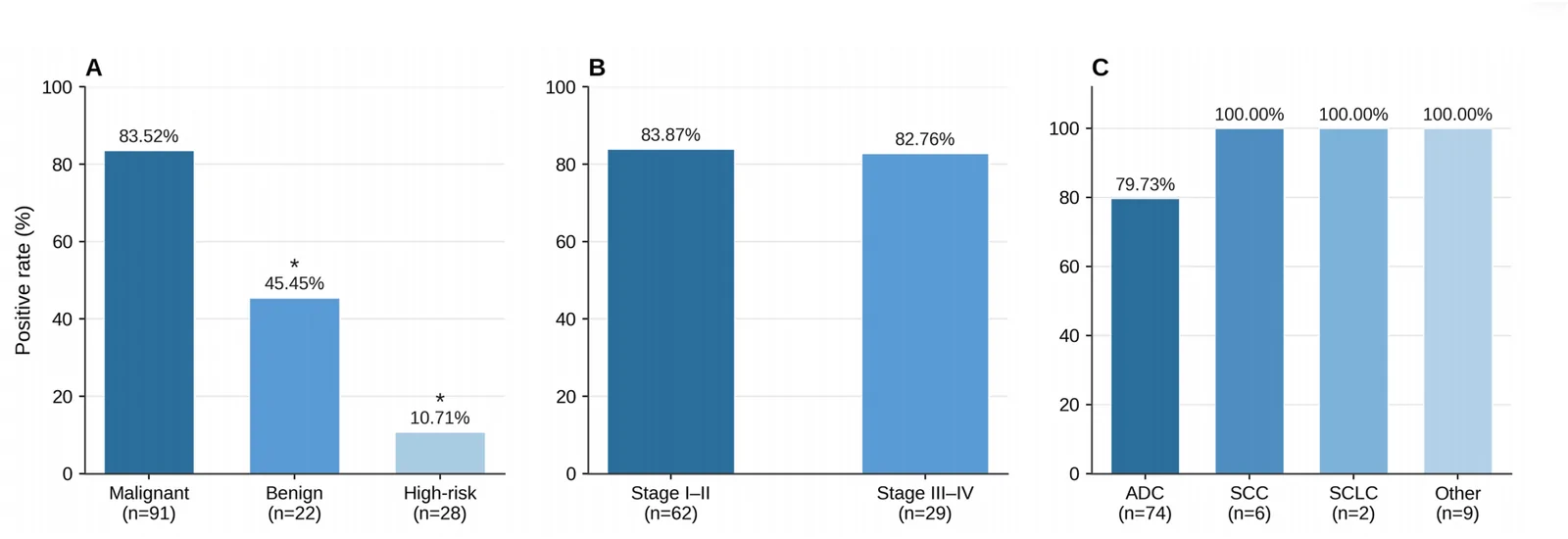
Positive rates of the three-DNA-methylation panel (plasma *SHOX2*, *RASSF1A*, and *PTGER4*) in the prospective methylation cohort. (A) Positive rate in the malignant, benign-nodule, and high-risk groups. (B) Positive rate in early-stage (I–II) versus advanced-stage (III–IV) malignant disease. (C) Positive rate by histological subtype among malignant cases. A sample was scored positive when at least one of the three markers exceeded its assay threshold. ADC, adenocarcinoma; SCC, squamous cell carcinoma; SCLC, small cell lung cancer. *P < 0.05 versus the malignant group.

The positive rate of the seven-autoantibody panel across all stages of malignant disease was 63.20% (122 of 193), significantly higher than in benign nodules (20 of 118; 16.95%) or the high-risk group (14 of 135; 10.37%) (*P* < 0.05; Fig. 2A). The positive rate did not increase with stage: 52 of 77 stage I–II cases (67.53%) versus 70 of 116 stage III–IV cases (60.34%) (*P* > 0.05; Fig. 2B). By histology, positivity was 71 of 112 adenocarcinomas (63.39%), 24 of 39 squamous cell carcinomas (61.54%), 26 of 40 small cell lung cancers (65.00%), and 1 of 2 other tumours (50.00%) (Fig. 2C). Positive rates by group and stage for all three assays are summarised in Table 2.

**Fig. 2:**
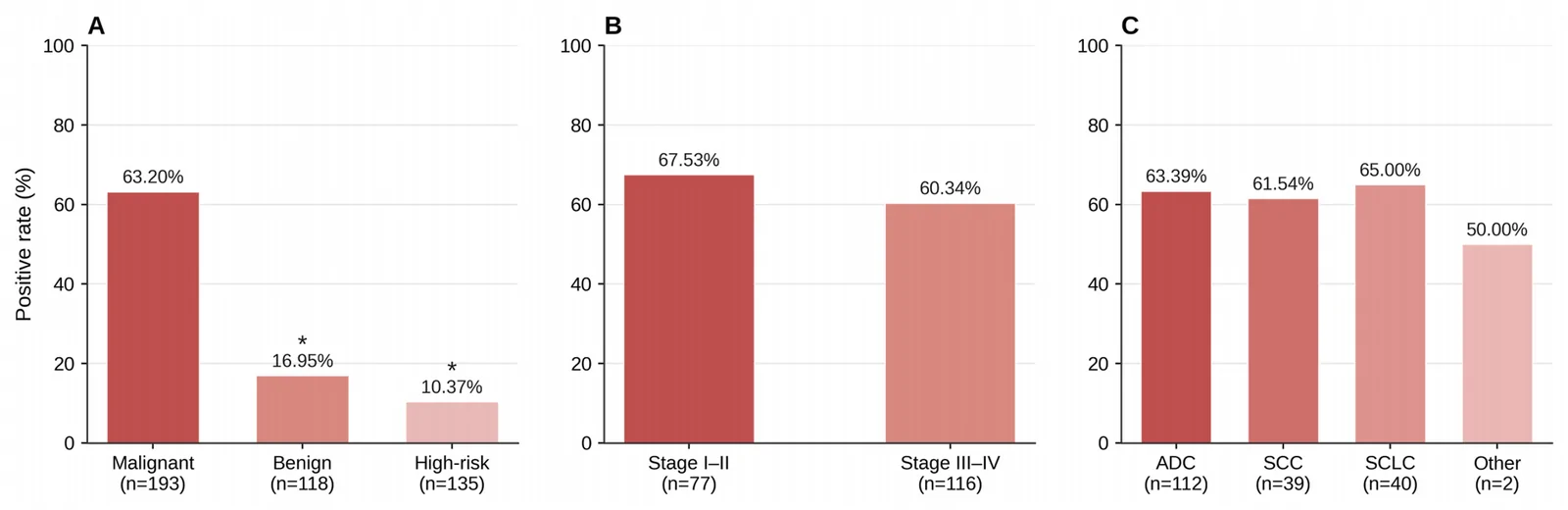
Positive rates of the serum seven-autoantibody panel (p53, GAGE7, PGP9.5, CAGE, MAGEA1, SOX2, and GBU4-5) in the retrospective autoantibody cohort. (A) Positive rate in the malignant, benign-nodule, and high-risk groups. (B) Positive rate in early-stage (I–II) versus advanced-stage (III–IV) malignant disease. (C) Positive rate by histological subtype among malignant cases. A sample was scored positive when at least one autoantibody exceeded its reference limit. ADC, adenocarcinoma; SCC, squamous cell carcinoma; SCLC, small cell lung cancer. *P < 0.05 versus the malignant group.

**Table 2:** Biomarker positive rate by group and stage.

Assay	Subgroup	Positive/total	Positive rate
Three-DNA-methylation panel	Malignant, all stages	76/91	83.52%
Three-DNA-methylation panel	Benign nodules	10/22	45.45%
Three-DNA-methylation panel	High-risk controls	3/28	10.71%
Three-DNA-methylation panel	Stage I–II malignant	52/62	83.87%
Three-DNA-methylation panel	Stage III–IV malignant	24/29	82.76%
Seven-autoantibody panel	Malignant, all stages	122/193	63.20%
Seven-autoantibody panel	Benign nodules	20/118	16.95%
Seven-autoantibody panel	High-risk controls	14/135	10.37%
Seven-autoantibody panel	Stage I–II malignant	52/77	67.53%
Seven-autoantibody panel	Stage III–IV malignant	70/116	60.34%
Conventional tumour markers	Stage I–II malignant	29/77	37.66%
Conventional tumour markers	Stage III–IV malignant	109/116	93.97%
Conventional tumour markers	Control groups	60/253	23.72%

### 3.3 Comparative diagnostic performance

We evaluated the ability of the two panels to distinguish lung cancer patients from benign pulmonary nodule controls. For early-stage malignant nodules or lung cancer, the methylation panel showed a sensitivity of 83.87%, specificity of 74.00%, AUC of 0.789, and Youden index of 0.579. The seven-autoantibody panel showed lower sensitivity but higher specificity, with a sensitivity of 67.53%, specificity of 86.56%, AUC of 0.771, and Youden index of 0.541. Conventional tumour markers had limited early-stage performance, with a sensitivity of 37.66%, specificity of 76.28%, AUC of 0.570, and Youden index of 0.139 (Fig. 3).

**Fig. 3:**
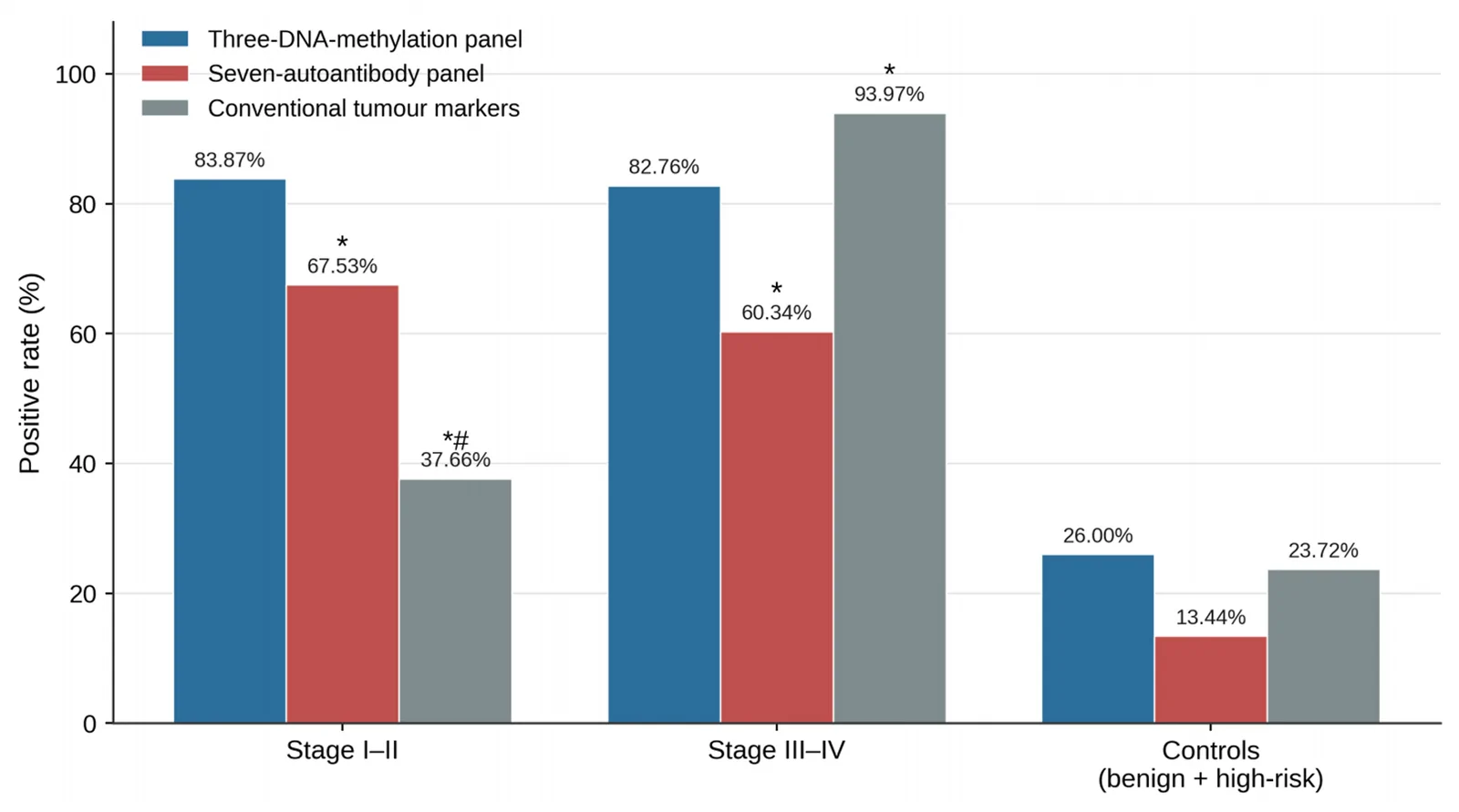
Comparative positive rates of the three-DNA-methylation panel, the seven-autoantibody panel, and conventional serum tumour markers (CYFRA21-1, CEA, NSE, and SCC) across disease stage and controls. In early-stage (I–II) disease the methylation panel had the highest positive rate, whereas conventional markers were highest in advanced-stage (III–IV) disease. Controls comprised benign pulmonary nodules and high-risk individuals. *P < 0.05 versus the three-DNA-methylation panel; #*P* < 0.05 versus the seven-autoantibody panel.

In stage III–IV disease, conventional tumour markers had the highest sensitivity (93.97%) and AUC (0.851), consistent with the greater tumour burden of advanced disease. The methylation panel retained similar sensitivity in late-stage (82.76%) and early-stage (83.87%) disease. Exploratory combined testing of the methylation panel and the seven-autoantibody panel improved early-stage sensitivity to 94.75%, but specificity decreased to 64.08%; the AUC was 0.794, only slightly higher than the methylation panel alone (Table 3).

**Table 3:** Diagnostic performance for malignant pulmonary nodules or lung cancer.

Assay	Stage	AUC	Sensitivity	Specificity	Youden index
Three-DNA-methylation panel	I–II	0.789	83.87%	74.00%	0.579
Three-DNA-methylation panel	III–IV	0.783	82.76%	74.00%	0.568
Seven-autoantibody panel	I–II	0.771	67.53%	86.56%	0.541
Seven-autoantibody panel	III–IV	0.735	60.34%	86.56%	0.469
Conventional tumour markers	I–II	0.570	37.66%	76.28%	0.139
Conventional tumour markers	III–IV	0.851	93.97%	76.28%	0.703
Methylation + autoantibody panel	I–II	0.794	94.75%	64.08%	0.588
Methylation + conventional markers	I–II	0.732	89.96%	56.44%	0.464
Autoantibody + conventional markers	I–II	0.728	79.69%	66.00%	0.457

Controls comprised benign pulmonary nodules and high-risk individuals. Combined early-stage analyses were exploratory. AUC, area under the receiver operating characteristic curve.

For all malignant cases versus all controls in the methylation cohort, the three-DNA-methylation panel produced a positive predictive value of 85.39% (76/89), negative predictive value of 71.15% (37/52), accuracy of 80.14% (113/141), positive likelihood ratio of 3.21, and negative likelihood ratio of 0.22. For stage I-II malignant disease versus all controls, the corresponding values were a positive predictive value of 80.00% (52/65), negative predictive value of 78.72% (37/47), accuracy of 79.46% (89/112), positive likelihood ratio of 3.23, and negative likelihood ratio of 0.22. These indices indicate that the three-gene methylation assay may be clinically useful for post-LDCT triage, particularly for maintaining sensitivity in early-stage disease.

## 4 Discussion

This study suggests that both plasma *SHOX2*/*RASSF1A*/*PTGER4* methylation and a seven-autoantibody panel have diagnostic value in the evaluation of pulmonary nodules. The methylation panel showed the highest sensitivity in stage I–II disease, whereas the autoantibody panel showed higher specificity. These findings support the concept that different blood biomarkers capture distinct biological signals: tumour-derived epigenetic alterations may improve early-detection sensitivity, whereas host autoantibody responses may provide a more specific but less sensitive signal.

The methylation results are consistent with prior studies of *SHOX2*, *RASSF1A*, and *PTGER4*. *SHOX2* methylation has been reported as a diagnostic biomarker in plasma and bronchial specimens, and panels combining *SHOX2* with other methylated genes improve lung cancer discrimination [[9], [10], [11], [12]]. *PTGER4* methylation in plasma also helps distinguish lung cancer from benign pulmonary nodules and chronic obstructive pulmonary disease [13]. The three genes capture complementary facets of the aberrant methylation programme that accompanies lung carcinogenesis. Hypermethylation of promoter-associated CpG islands is one of the earliest and most frequent molecular events in tumour development, often preceding detectable driver mutations and clinically evident disease [7,8]. *SHOX2*, a homeobox transcription factor on chromosome 3q, shows recurrent CpG-island hypermethylation in lung tumours; although its precise role as a driver is still being defined, methylated *SHOX2* is a robust and reproducible marker of malignancy that can be measured in plasma and bronchial specimens [9,10]. *RASSF1A*, in the 3p21.3 tumour-suppressor locus, is a canonical tumour suppressor whose promoter hypermethylation silences a protein involved in cell-cycle control, microtubule stability, and apoptosis; its inactivation is common across non-small cell and small cell lung cancer and contributes to genomic instability [7,11]. *PTGER4*, which encodes prostaglandin E receptor 4, undergoes hypermethylation that discriminates lung cancer from benign nodules and COPD independently of tumour volume, smoking status, and age [13].

Because no single locus is methylated in every tumour, and because different histological subtypes and individual tumours carry different methylation patterns, combining the three markers increases the proportion of cancers detected while each marker retains high specificity in non-malignant controls. This complementarity explains why a *SHOX2*/*RASSF1A*/*PTGER4* panel achieves higher and more uniform sensitivity across adenocarcinoma, squamous cell carcinoma, and small cell lung cancer than any single marker [12], and it is consistent with the near-complete positivity we observed in the non-adenocarcinoma subgroups, albeit in small numbers. Critically, because these methylation changes arise early, the panel retained high sensitivity in stage I–II disease (83.87%), comparable to that in stage III–IV disease (82.76%). This stage-independent behaviour is the property most relevant to post-LDCT nodule triage, where the clinical priority is to identify early, potentially curable malignancy [7,12].

The autoantibody results also agree with previous work. Systematic reviews and Chinese population studies have shown that tumour-associated autoantibody panels can aid lung cancer detection, although their sensitivity is generally modest [15,16]. A prior study from our group found that a seven-autoantibody panel combined with the Mayo model improved differentiation of pulmonary nodules [17]. The ECLS randomised trial further showed that autoantibody testing followed by imaging could shift diagnosis toward earlier stages in a screening context [18]. Routine-practice audit data and more recent clinical studies likewise indicate that seven-autoantibody testing can contribute to lung cancer risk stratification when interpreted in the appropriate clinical setting [19,22].

Tumour-associated autoantibodies arise when the host immune system recognises antigens that are aberrantly expressed, mutated, over-expressed, or misfolded in tumour cells, and the resulting antibodies can be comparatively abundant and stable in serum even when the corresponding antigen is present at low levels [15,16]. The seven antigens in this panel sample distinct oncogenic processes: p53 accumulates when the *TP53* tumour-suppressor gene is mutated; SOX2 is a transcription factor frequently amplified in squamous cell and small cell lung cancer; MAGEA1, GAGE7, and CAGE are cancer–testis antigens that are silenced in most normal somatic tissues but re-expressed in tumours; PGP9.5 (also known as UCHL1) is a neuroendocrine ubiquitin hydrolase over-expressed in lung cancer; and GBU4-5 is a helicase antigen. Because any individual autoantibody is elicited in only a subset of patients, single markers have limited sensitivity; aggregating seven complementary specificities captures a larger fraction of tumours and raises overall sensitivity while preserving the high specificity characteristic of autoantibody assays [15,17]. The differing tissue origins of these antigens also underlie the relatively even positivity we observed across adenocarcinoma, squamous cell carcinoma, and small cell lung cancer [17,22]. Because the humoral response to tumour antigens can develop before a nodule becomes radiographically conspicuous, the panel retained useful sensitivity in stage I–II disease and, in the ECLS trial, shifted the stage distribution of screen-detected cancers toward earlier, more treatable disease [18]. In the present cohort the specificity of the autoantibody panel exceeded that of DNA methylation, which is clinically valuable when limiting false-positive results and unnecessary invasive procedures is the priority.

Conventional serum tumour markers behaved differently. Their early-stage sensitivity was low, but their late-stage sensitivity was high. This pattern is biologically plausible because circulating protein marker levels often rise with tumour burden, advanced stage, or specific histological subtypes. Conventional markers may therefore remain useful for advanced-disease assessment but are not sufficient as standalone early diagnostic tools.

Combined testing increased early-stage sensitivity but reduced specificity. The AUC gain for the methylation panel plus the autoantibody panel was small compared with the methylation panel alone. Combination testing may therefore be most useful in clinical pathways where missing early lung cancer is the dominant concern, but it should not replace imaging interpretation, clinical risk models, or tissue diagnosis when malignancy remains suspected. Future models should integrate biomarkers with LDCT features, Lung-RADS or other structured imaging scores, smoking exposure, age, and nodule size, density, and growth. Other blood-based nodule classifiers, including the PANOPTIC plasma proteomic classifier, also support the value of integrating biomarkers into post-imaging nodule pathways rather than using blood tests as isolated screening tools [23].

A direct head-to-head comparison with clinically available imaging techniques could not be performed in the present retrospective/prospective dataset because standardized Lung-RADS categories and complete CT risk-model variables were not recorded for every participant. We have therefore framed the methylation panel as an adjunctive molecular test and not as a substitute for standard-of-care imaging. Future validation should prospectively compare LDCT/risk-model assessment alone, methylation alone, and integrated imaging-plus-methylation models using prespecified decision thresholds.

Common actionable genomic alterations, including EGFR mutations and ALK, ROS1, BRAF, MET, RET, NTRK, KRAS, and ERBB2 alterations, are essential for treatment selection in confirmed lung cancer [24]. However, these markers were not systematically tested in benign nodule or high-risk control participants and were generally assessed after pathologic diagnosis rather than at the time of pre-diagnostic nodule triage. Adding tumour-only actionable marker status to the present diagnostic model would therefore introduce selection and verification bias. Prospective studies using plasma-based genomic profiling may evaluate whether cfDNA mutation data improve the accuracy of methylation-based diagnosis, particularly when tissue is limited [25].

This study has limitations. First, the methylation cohort was relatively small, especially for benign nodules, high-risk controls, and non-adenocarcinoma histological subgroups. Second, the methylation and autoantibody analyses were conducted in different cohorts, which limits direct patient-level comparison between assays. Third, the study population was drawn from hospitals in a single region, and external validation is required before broad clinical application. Fourth, the combined analyses were exploratory and should be confirmed using prospectively collected patient-level data with prespecified decision thresholds. Finally, cost-effectiveness, downstream biopsy rates, and patient-centred outcomes were not evaluated.

The three-gene design also has biological limitations. No single methylation locus is universally altered across all lung cancers, and methylation signal strength can vary by histology, tumour volume, vascular shedding, necrosis, and the amount of tumour-derived cfDNA in plasma. Benign inflammatory or infectious lung disease may also generate background molecular noise. Broader methylation signatures or multi-omic models that combine methylation with CT features, clinical risk factors, autoantibodies, protein markers, or cfDNA mutation data may improve sensitivity and specificity, but such models require larger training and independent validation cohorts to avoid overfitting.

## 5 Conclusion

Plasma *SHOX2*/*RASSF1A*/*PTGER4* DNA methylation and the seven-autoantibody panel both demonstrated value for differentiating benign and malignant pulmonary nodules. DNA methylation provided stronger early-stage sensitivity, whereas the autoantibody panel provided higher specificity. A combined testing strategy may improve early-stage sensitivity, but the modest AUC gain and reduced specificity indicate that larger multicentre validation and integration with clinical–imaging models are needed before routine implementation.

## Ethics approval

The study was approved by the Ethics Committee of Liuzhou Workers' Hospital (KY2025472 and KY2024005).

## Data availability

Deidentified study data may be made available from the corresponding author subject to institutional approval.

## Author contributions

Dong Rui, Xunsi Liang, Min Yi, Shaowei Zhan, Shui Xiang, Jifei Chen, Jiachen Hou, Xin Wu contributed to data collection, statistical analysis, and manuscript drafting. Xuejun Qin and Zhougui Ling contributed to study conception, supervision, methodology, and critical revision of the manuscript. Both authors read and approved the final manuscript.

## Ethics declaration

Written informed consent to take part in the study and to publish the article has been obtained from all participants or their legal representatives. The privacy rights of participants have been observed.

This study included organ or tissue donors. This study includes human biological material and consent was obtained by donors, or their next of kin or legal representatives, for use in this study and for publication of the article. The samples used in this research were not sourced from executed prisoners or prisoners of conscience.

This study was performed in compliance with relevant laws, regulatory frameworks and guidelines where the research took place. This study was approved by the Ethics Committee of Liuzhou Workers' Hospital. (Approval No. KY2025472,KY2024005)

This research follows the CARE guidelines and the CARE checklist.

## Funding

This work was supported in part by the 10.13039/501100017691Key Research and Development Program of Guangxi Zhuang Autonomous Region (No. FN2600640007), in part by the Guangxi Natural Science Foundation (No. 2026GXNSFAA00640165),in part by the Liuzhou Science and Technology Programs (No. 2024SB0105A003; No. 2024YB0103B002), and in part by the Guangxi Medical and Health Key Discipline Construction Project.

## Declaration of competing interests

The authors declare that they have no known competing financial interests or personal relationships that could have appeared to influence the work reported in this paper.
